# Copolymerization of pyrrole and carbazole onto polyester textile

**DOI:** 10.55730/1300-0527.3644

**Published:** 2024-01-18

**Authors:** Mehmet Akif KÜÇÜKKAYA, Utku USTAMEHMETOĞLU, Esma SEZER, Belkıs USTAMEHMETOĞLU

**Affiliations:** 1 Department of Chemistry, Faculty of Science and Letter, İstanbul Technical University, İstanbul, Turkiye; 2 Department of Manufacturing, Faculty of Mechanical Engineering, İstanbul Technical University, İstanbul, Turkiye; 3 Devinno Technology Investments Inc.

**Keywords:** Polypyrrole, polycarbazole, copolymer, polyester, conductive textile composites

## Abstract

The purpose of this study was the polymerization of carbazole (Cz), pyrrole (Py) and copolymerization of them onto polyester (PES) textile with chemical oxidative method by using FeCl_3_. First, in order to determine the optimum conditions, the effect of polymerization steps, the immersion order of the PES to the oxidant and monomer solutions, time, monomer, oxidant, and surfactant concentrations and types on the conductivities of PES/PCz composite were investigated and at this conditions, conductive composite, PES/P[Py-co-Cz] was obtained. The highest conductivities were obtained as 12.4 mS/cm and 9.0 mS/cm for PES/PCz and PES/P[Py-co-Cz], respectively. Further characterization of PES/P[Py-co-Cz] was performed by conductivity, FTIR, scanning electron microscopy (SEM), and dynamic mechanical analysis (DMA) measurements, and results were compared with PES/PCz and also PES/PPy. The presence of Py and Cz in the same polymer chain created synergy and improved the conductivity and mechanical properties of the composite.

## 1. Introduction

In recent years, with the expansion of areas of conductive polymers (CPs), they have become an important alternative to metals. Nowadays it is known that a large number of CPs like polypyrole (PPy), polycarbazole (PCz), polyaniline (PANI), polythiophene (PTh), polyfuran (PFu), polyethylenedioxythiophene (PEDOT) can be used in various fields such as electroluminescence, microelectronics, textiles, invisible aircraft dye semiconductor chips, integrated circuits, solar cells, light battery components, sensors, antistatic coating, packaging, transistors, screens, sun panels, heat generation electromagnetic shielding, the light emitting devices (LEDs) and sensors [1–2]. However, their weak mechanical properties and insolubility limit their capability to be shaped for the intended purpose [3]. As a method to overcome this problem, CPs are coated onto flexible carriers [4].

Recently, textiles have played a significant role in various engineering applications involving polymers, ceramics, and metals. With the rapid developments in the electronics industry, the demand for flexible, conductive, and semiconductor materials is increasing.

A conductive textile refers to a fabric capable of transmitting electricity, with the spraying of CPs, filaments can be formed or weaving it to make a textile or other different methods can be used to make a monomer polymerize onto an insulating textile to form a conductive homogeneous layer. This, with the wide range of structural and mechanical properties of the textile allows the combination of electrical properties of the CPs [5]. The thickness of the conductive film depends on the synthesis time of the monomer and the oxidant concentration, it is valued in microns. These (textile/CP) composites present several processing challenges that need to be overcome to produce pure forms of CPs, and these powerful, flexible, and sometimes elastic conductive composites find extensive usage in various applications [6–8]. Because PPy’s excellent adhesion sticks very well to different bearing surfaces, it is the most commonly used polymer to prepare textiles that have electrical conductivity and it may form a composite with insulating fibers or fabric [9]. Previous studies have reported the use of silk [10], wool [11], PES [12], cotton [13], and nano cellulose [14] were used with PPy to form conductive textiles. PTh and its substituents [15–16] and PANI [17] can be considered as the other most used polymers to form conductive textiles. On the other hand, while PCz has been extensively studied for its good electronic and electrochromic properties [18,19], no article has been found discussing its application in conductive textiles.

To obtain a copolymer, two different monomers must be polymerized in the same reaction medium. In literature, various copolymers were coated with different methods on different textiles, and the surface resistivity of some of them was reported. The best methods for coating monomer mixtures or two monomers chemically bonded to each other (comonomer) as a thin film on textiles are chemical and electrochemical polymerization [20], treatment with chemical vapor [21], and electrochemically blending conductive textile solutions [22]. In literature, Py and thiophene (Th) were copolymerized onto PES textile by chemical polymerization and its resistance was reported as 50 kΩ [23].

In another study, in situ deposition of PANI and PPy electroconductive layers on textile surfaces by the reactive ink-jet printing technique was reported [24]. PEDOT and p-toluenesulphonic acid (PTSA) copolymer were coated on PES textile and the surface resistivity was reported as 10 Ω/sq [25]. Textiles that have cotton and PES in different ratios were made into a stretchable electrochromic textile by dipping the textile into PEDOT-polystyrene sulfonate (PSS) solution, and a composite that has 350 Ω was made [26].

In our previous study, PCz was chemically coated on PES textile starting from Cz and CAN, and the synthesis and characterization of conductive composite, PCz/PES was reported [27].

In this study, copolymer of Py and Cz onto PES textile (PES/P[Py-co-Cz]) was synthesized for the first time under different monomer concentrations and FeCl_3_ as oxidant. The optimum conditions were determined from the data obtained by conductivity measurements. Characterization suggested that PES/P[Py-co-Cz] has better mechanical properties than PES/PPy and PES/PCz that were synthesized under the same conditions.

## 2. Experimental

### 2.1. Materials

Pyrrole (Py), Carbazol (Cz), dodecyl benzene sulfonic acid (DBSA), para toluene sulphonic acid (PTSA), Na_2_CO_3_, Sigma Aldrich, FeCl_3_, cerium (IV) ammonium nitrate (CAN), acetonitrile (ACN) and toluene (Carlo Erba), were used in analytical grade and no purification was made. PES used in the project is 100% polyester fabric with 0.25 mm thickness.

### 2.2. Measurements

Resistance of the conductive materials is generally measured using the four-probe technique. However, due to the fibrous structure of the textile material, a prop is used consisting of two disks instead of a four-point probe technique. The electrical resistance of the conductive textile obtained in this study with the help of two disks is measured with a GWINSTEK LCR meter device. The experiments were carried out twice, 5 measurements were taken from the upper and lower surfaces of the same sample, and average values were given and converted to conductivities by using the thickness of coated textiles. The percentage error was determined as 3%. Surface morphologies of copolymer-coated textile samples were obtained by using Carl Zeiss-EVO MA-10 brand scanning microscope (SEM), mechanical properties, using TA Q800 DMA, the structural features, using Perkin-Elmer ATR- FTIR Spectrum One Spectrophotometer based on measurement of the reflected light from the materials.

### 2.3. Preparation of PES/PCz, PES/PPy and PES/P[Py-co-Cz]

Various methods have been reported for the preparation of conductive textiles by chemical polymerization, one of which is coating the textile by first immersing it in a monomer solution and then in an oxidant solution [28]. The aim of all methods is to obtain a fully CP-coated textile surface instead of polymerization in solution by adjusting conditions to slow down the reaction. In this study, a method used in our previous studies and known to be effective was applied [29]. Various concentrations were used for both monomer and oxidant and optimum conditions were determined by sequentially immersing the textile in monomer and oxidant solutions.

The effect of immersion of textile in different solutions and polymerization time on the conductivities of PES/PCz was investigated and changes in conductivities with different polymerization procedures are given in Table 1.

In the first experiment, the textile sample was directly immersed in Cz solution for 12 h and then in FeCl_3_ solution for 12 h (Table 1, procedure A). However, sufficient i.e. homogeneous, stable, and conductive polymer on textile was not obtained in this experiment. Then, in order to understand the effect of the wetting process on polymerization, before the polymerization, to remove dirt and oil, that came from the production, and soils on the textile, firstly it was waited in 1 g/L aqueous detergent solution containing 1 g of Na_2_CO_3_ at 70 °C for 30 min. In addition, in the literature, the effects of noncationic, nonanionic, and nonionic surfactants on the synthesis of conductive textiles were investigated and they suggested that the presence of surfactants contributes to the uniform coating of CPs on textile surface [30,31]. Therefore, in this study, DBSA and PTSA were tried as wetting agents and DBSA was chosen as a surfactant during synthesis and the effective concentration was determined as 0.02 M.

During pretreatment of the PES surface, in order to absorb Cz better, a certain weighted PES cloth was placed in the aqueous solution I (0.02 M DBSA + 15 mL water) and then waited in the ACN solution II (0.02 M DBSA + 15 mL ACN separately, both for 30 min (Procedure B).

Chemical polymerization was performed in separate solutions namely, solution III (0.01M Monomer + 15 mL ACN) and IV (0.6 M FeCl_3_ + 15 ml ACN) by waiting for PES fabric for an hour in order to ensure that the textile surface is absorbing the monomers and oxidant. In the next step, PES with absorbed monomers on was waited for FeCl_3_ solution in ACN containing DBSA (IV), for 10 min to oxidize monomer. Since the polymerization reaction is fast, in order to increase the yield of PCz on the PES surface instead of the solution, the polymerization was carried out by immersing the PES in solutions III and IV several times successively. The dark green colored textile, after the sequence of immersing in III and IV is repeated 8 times. They were washed with ACN, dried at room temperature, weighted and it was kept under standard conditions until it was dried, 16 h,

With increasing duration of polymerization from 270 min to 540 min, the conductivity decreases (Table 1, procedure C) and the optimum condition for obtaining PES/PCz was determined through experimental studies as procedure B and used for also PES/PPy.

**Scheme 1:**
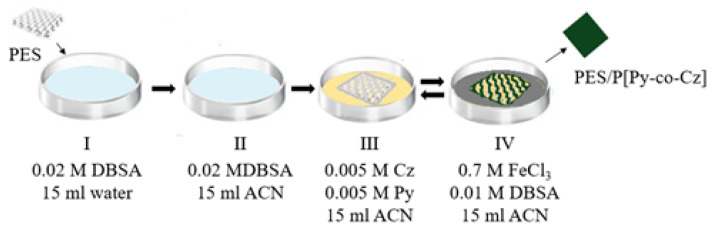
Preparation steps of the PES/[PPy-co-Cz] which provides optimal conditions for the solution used to wet the PES textile (I, II) and the polymerization reaction takes place (III, IV).

In order to obtain PES/P[Py-co-Cz], procedure B was used during the copolymerization of Py and Cz on PES. Polymerization conditions are summarized in Table 2 and the schematic representation of the polymerization steps at the optimum condition is shown in Scheme 1.

PES/PCz and PES/P[Py-co-Cz] were weighted and the percentage yield (Y%) was calculated from the difference between the weights of raw PES (w_0_) and coated PES (w) (Equation 1).


(1) $$\text{Y}\%=(\text{w}-{\text{w}}_{0})/\text{w}$$

In equation 1, w_0_ is the weight of the raw textile, and w is the weight of the PES/P[Py-co-Cz] after the copolymerization.

## 3. Results and discussions

### 3.1. The effect of oxidant and solvent types on the properties of PES/PCz

It is important to determine the type and amount of oxidant in obtaining conductive polymers with the desired properties, and as suggested in the literature [32], it has been observed that the use of oxidants with high oxidation potential and high amounts causes cross-linking and increased resistance. On the other hand, using a small amount of oxidant causes less coating of the textile.

**Figure 1:**
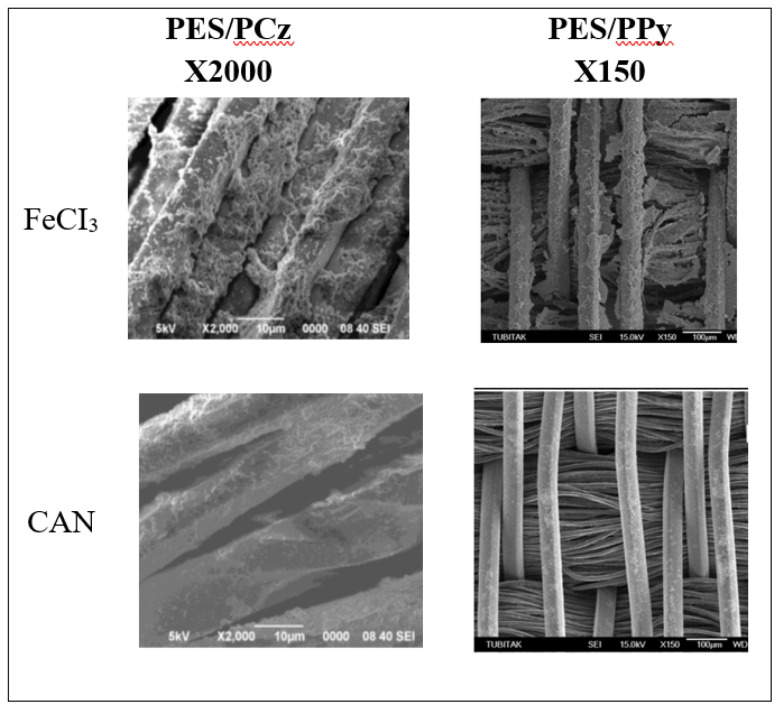
Comparison of SEM images of PES/PCz and PES/PPy obtained by FeCl_3_ and CAN.

Polymerizations were carried out using both FeCl_3_ and CAN as oxidants, and the SEM images of the obtained PES/PCz and PES/PPy are presented in Figure 1. Although the magnifications of PES/PPy are different than the images of PES/PCz, in the case of CAN, due to having high oxidation potential, both in the cases of PCz or PPy could not accumulate on the textile and polymerization occurred in solution. FeCl_3_ with lower oxidation power has been chosen to slow down the oxidation rate and provide polymerization on the substrate.

Aprotic solvents such as acetonitrile, benzonitrile, and dichloromethane are preferred due to their weak nucleophilic character so that the cation radical formed during the synthesis of conductive polymers in solution, on the metal or flexible substrate surface, reacts more easily [1,33,34]. In addition, solvents such as toluene and propylene carbonate are also used to provide flexibility to brittle conductive polymers [35,1]. In order to increase the solubility of the supporting electrolyte and therefore the conductivity of the solution, polymerizations are also carried out in aqueous environments by adding water to the organic solvent and suitable monomers [33].

On the other hand, PES is generally resistant to organic solvents but perchloro ethylene tetrachloro ethylene, methylene chloride, trichloro ethylene, hexafluoro propanol, phenol/tetrachloroethane, trichloroacetic acid/chloroform or alkylene carbonates dissolve PES [36].

In light of this information, polymerization of Cz on the PES surface was carried out by using water, toluene, ACN, and ACN+water mixtures to determine the appropriate solvent in this study and conductivities of resulting PES/PCz, were measured (Table 3). ACN was chosen as the solvent, similar to the literature [29,33,34] since the highest conductivity values were obtained when polymerization was performed by using ACN both for Cz and FeCl_3_.

### 3.2. The effect of monomer and oxidant concentration on the properties of PES/PCz and PES/P[Py-co-Cz]

**Figure 2:**
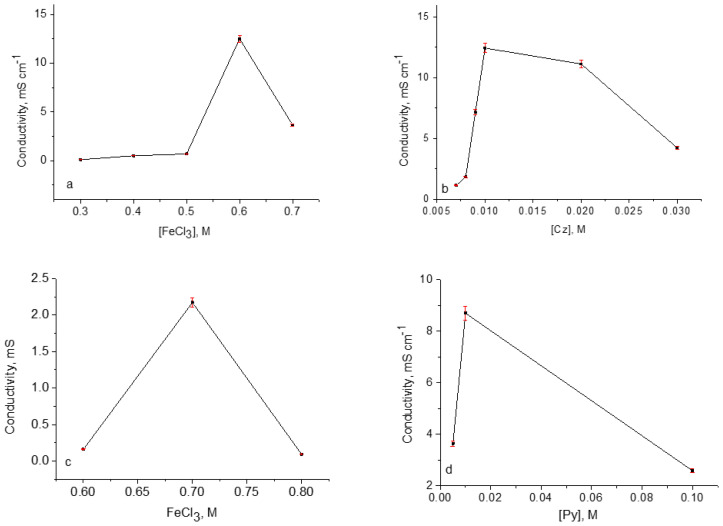
The variation in the conductivity values of PES/PCz with different FeCl_3_ concentrations (a) of PES/PCz with different Cz concentrations. (b) PES/P[Py-co-Cz] with different FeCl_3_ concentrations (c) PES/P[Py-co-Cz] with Py concentration, [Cz] = 0.01 M and [FeCl_3_] = 0.7 M (d).

Since PES is an insulator, the surface resistance is expected to decrease when coated with a conductive polymer. PES/PCz on the PES surface was obtained at different concentrations of FeCl_3_ as well as different concentrations of Cz, the polymerization yields were calculated according to Equation 1 and summarized in Table 4. The conductivity values were obtained as suggested in the literature [37] and plotted versus the variation of concentrations of FeCI_3_ (Figure 2a), and Cz (Figure 2b), in order to determine the optimum chemical polymerization conditions of Cz. As can be seen when the FeCl_3_ concentration increases up to 0.6 M, the conductivity increases. This behavior can be explained by PCz creating a layer on the PES surface and its surface resistance decreases. After the concentration of 0.6 M FeCl_3_, reactions such as carbonyl formation that disrupt the conjugation of the Cz rings in the polymer chain due to excessive oxidation or cross-linking of the chains cause a decrease in conductivity, as suggested for similar studies in the literature [30]. As seen in Figure 2b, the conductivity of the PES/PCz was firstly increased with the increase in Cz concentration and when the molarity of Cz is more than 0.01 M, it started to decrease. This behavior can be explained by unreacted Cz, which is insulating.

PES was coated with copolymer of Cz and Py by using different concentrations of monomers and FeCI_3_, the conductivity of resulting PES/P[Py-co-Cz] was measured and results were given in Figures 2c and 2d. It is observed that when the FeCl_3_ concentration increases, the conductivity increases up to 0.7 M (Figure 2c). This behavior can be explained by the decrease in surface resistance as the P[Py-co-Cz] layer forms on the PES surface. After 0.7 M FeCl_3_ concentration, overoxidation occurred due to excess FeCl_3_ and carbonyl groups formed on the Py or Cz rings of the copolymer chain, which disrupted the conjugation of the chain [32,38]. Another explanation is the formation of cross-links between the chains. According to this result, FeCl_3_ concentration was chosen as 0.7 M for PES/P[Py-co-Cz].

The effect of the mole ratio of the Cz and Py, n_Cz_/n_Py_ on the conductivity was investigated. First Cz concentration stayed constant (0.01 M) and n_Cz_/n_Py_ was changed according to Py concentration (Figure 2d). As it can be seen, with the increase in Py concentration up to 0.01 M, the conductivity of the PES/P[Py-co-Cz] was first the conductivity increased and then it started to decrease. This behavior can be explained as the increase in Py concentration after the optimum mole ratio (n_Cz_/n_Py_ = 1) and since the Py monomer has higher reactivity than Cz [18,37], Py reacts rapidly with FeCl_3_ in the solution instead of the PES surface, and the formation of less polymer on the surface causes a decrease in conductivity.

**Figure 3:**
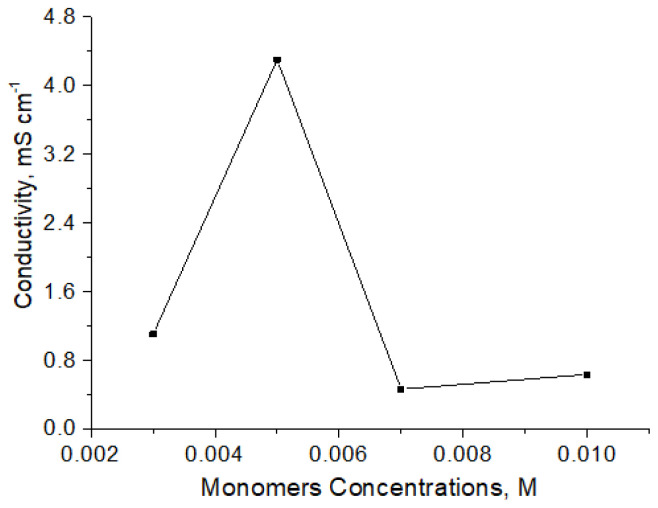
The variation in the conductivity values of PES/P[Py-co-Cz] with increase in the amount of Py and Cz concentrations at constant mol ratio (n_Cz_/n_Py_ =1), [FeCl_3_] = 0.7 M.

The occurrence of polymerization in solution or on the PES surface depends on the oxidant type and concentration, as well as on the monomer concentration. When the monomer concentration is high, polymerization may occur in solution rather than on the textile surface, if it is low, the amount and conductivity of the polymer obtained on the surface will be very low, since a sufficiently high conjugation length cannot be achieved. For this reason, experiments were carried out by changing the Py and Cz concentrations (0.003 M, 0.005 M, 0.007 M, 0.01 M) at constant mol ratio (n_Cz_/n_Py_ = 1) and the conductivity of the PES/P[Py-co-Cz] formed was measured (Figure 3).

The optimum concentrations were determined as 0.005 M for both monomers. This result showed that under the condition that the FeCl_3_ concentration was kept constant at 0.7 M, it was possible to coat the textile with PES/P[Py-co-Cz] when the monomer concentrations were reduced enough to slow down the reaction rate. The concentration below this optimum value is not sufficient for the coating, and at higher concentrations, polymerization occurs in the solution instead of on the textile surface.

### 3.3. SEM results

**Figure 4:**
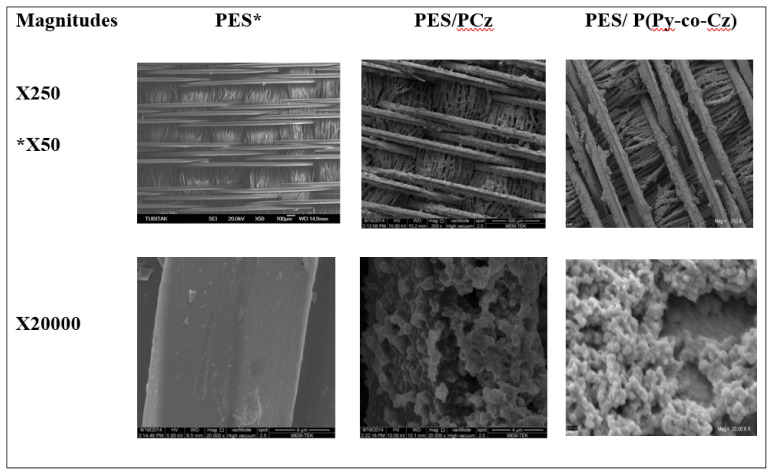
SEM images of PES, PES/PCz, and PES/P[Py-co-Cz] under different magnifications.

The SEM images of the PES, PES/PCz, and PES/P[Py-co-Cz] are presented in Figure 4. In previous studies [18,37–40], in the SEM images of the PPy and PCz obtained in the solution showed characteristic cauliflower appearance of PPy and smaller granular structure of PCz, together. When the polymerization is done on PES surface instead of the solution, the same granular structure is observed to be accumulated on the textile. In the SEM image when the magnification of 250 the uncoated textile weave structure is smooth and distinct appearance (Figure 4) similar to literature [34]. If the PES surface is coated with PCz and P[Py-co-Cz], it is seen that the places between the fibers are partially filled with polymer, and the existence of the polymer is seen clearer if the magnification increases to 20,000. In the case of PES/P[Py-co-Cz], the surface of PES seems to be covered by a much granular structure of the polymer film in the entire textile surface than in the case of PES/PCz. This is probably due to the effect of the different reaction rates of starting monomers. Similar comments have been made in the literature [34] for SEM images of PPy-coated PES with different dopant anions.

### 3.4. FT-IR results

**Figure 5:**
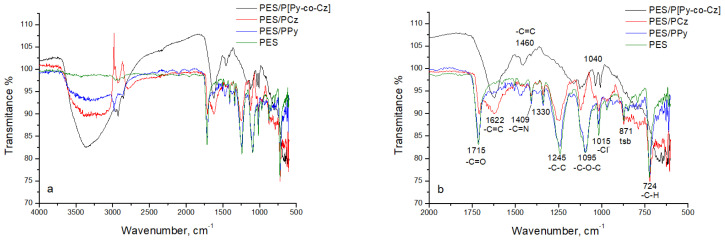
The FT-IR spectra of PES, PES/PCz, PES/PPy, and PES/P[Py-co-Cz] in the range of 4000–500 cm^−1^ (a) and 2000–500 cm^−1^ (b).

FT-IR spectra of uncoated PES, PES/PPy, PES/PCz, and PES/P[Py-co-Cz] are given in Figure 5a. The wavelengths of the peaks were indicated an enlarged spectrum. In the FT-IR spectrum of PES, it is seen that there are characteristic peaks of -C=O stretching at 1708 cm^−1^, -C-C stretching at 1240 cm^−1^, -C-O-C str. at 1097 cm^−1^ and -C-H bending at 720 cm^−1^ [41] (Figure 5b). In the spectra, the peaks at 871 cm^−1^ belong to 1,2,4 three substituents benzene (tsb), at 1015 cm^−1^ to dopant ion, Cl^-^ [42], at 1409 cm^−1^ to -C=N str, at 2983 cm^−1^ to -C-H str and at 3360 cm^−1^ to -N-H str.

In the case of PES/PPy, the -C-H out of plane deformations vibrations in the five membered carbon structure of Py ring, has been observed at 933 cm^−1^ and 966 cm^−1^ which absence in the case of PES/PCz composite. In the case of PES/P[Py-co-Cz], the shift of the wavelength of this peak to 1005 shows that the Py ring takes part in the copolymer structure.

Although the peak at 1240 cm^−1^ is observed in the case of PES, PCz, and PPy, it is absent in the case of P[Py-co-Cz]. Similarly, the peak intensity of the peak at 1095 cm^−1^ is stronger than in the cases of PPy and PES, than PCz, and the smallest in the case of P[Py-co-Cz]. This result is related to polymer layer thickness on the PES since the measurement carried out the reflectance mode of FT-IR and the decrease of polymer layer thickness results in an increase of the intensity of the characteristic peaks of PES.

On the other hand, the characteristic -C=O str. peak of PES at 1708 cm^−1^ was observed both for PES/PCz and PES/PPy. The reason for the lower intensity of this peak is in the case of PCz than in the case of PPy can be explained by the fact that the polymerization rate of Cz is lower than that of Py, so more polymer is formed on the surface instead of the solution which makes difficult the observation of the characteristic peak of PES. In the case of PES/P[Py-co-Cz], this peak is observed as a broad peak together with the -C=C str peak at 1622 cm^−1^ which corresponds to the aromatic -C=C str of PCz. On the other hand, the presence of peak observed both the spectra of PES/PPy and PES/P[Py-co-Cz] at 1460 cm^−1^ that corresponds to C=C str. of Py ring supports the inclusion of Py to the copolymer structure. This result may be due to the fact that, in the case of PES/PPy or PES/PCz, the PPy or PCz layers covering the PES surface are thin enough to see the characteristic peak of PES, but the P[Py-co-Cz] layer on the PES surface is too thick to detect this characteristic peak separately. These results are also in agreement with SEM image results and support the formations of PES/PPy, PES/PCz, and PES/P[Py-co-Cz]. When the peak areas at 1460 cm^−1^ in PES/PPy and the copolymer, and at 1622 cm^−1^ in PES/PCz and the copolymer were compared, it can be concluded that Py units are incorporated into the copolymer structure approximately 4 times larger proportion than the Cz units.

### 3.5. Copolymerization Mechanism of Py and Cz on the PES surface

In the previous study [37], when Py and Cz monomers oxidizes with CAN in solution, it was reported that cation radicals of Cz and Py react and P[Py-co-Cz] was formed.

According to IR and SEM results, although the polymerization mechanisms are the same, the copolymer film obtained on the PES surface is thicker than the PCz film and is preferably on the textile surface instead of solution due to the higher oxidation potential of Cz and significantly lower reactivity of radical cations of Cz than that of Py as suggested for Py-Th copolymer in the literature [43].

**Scheme 2:**
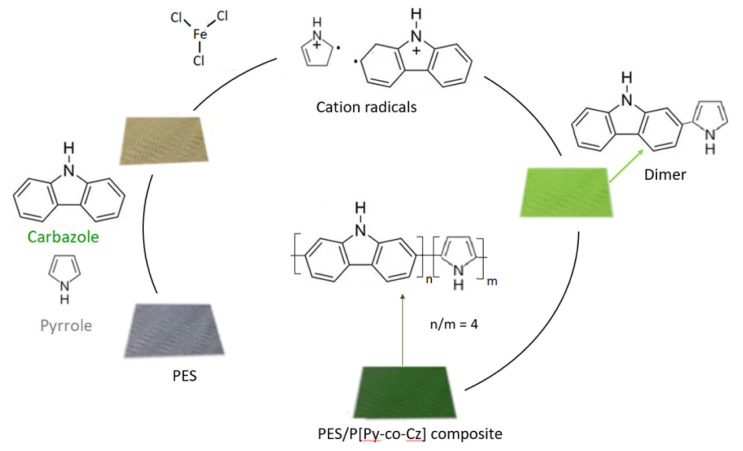
Formation of the radical cations of Py and Cz and mechanism and structures of PES/P[Py-co-Cz] on the PES surface.

IR results of this study showed that Py units are generally incorporated into copolymers in larger proportions than the Cz units. Although according to the elemental analyze result of a P[Py-co-Cz] obtained in our previous study [37], the mole ratios of Cz to Py in copolymer structure was found as 1/3, in the case of PES/P[Py-co-Cz] this ratio became 1/4. The reason for this difference can be explained by slowing down the reaction by using FeCI_3_ instead of CAN and this helps more deposition of PPy on the PES surface instead of the solution. In light of this information, the copolymerization mechanism can be suggested as shown in Scheme 2.

### 3.6. Mechanical analysis results

Conductive textiles are suitable materials for use in flexible device applications such as supercapacitor, gas, and biomechanical sensors [34], In our previous study, the DMA measurement of PES and PES/PCz were performed [27]. In this study, DMA measurements of PES/PPy and PES/P[Py-co-Cz] were carried out and the results are summarized in Table 5 in comparison with PES and PES/PCz.

A high Young’s modulus of a material indicates that it is difficult to stretch elastically, while a low value indicates that it can be stretched elastically relatively easily. As can be seen, when PES is coated with PPy, the module that indicates the flexibility of the textile, decreases compared to PES. This might have been because of the rigidity of PPy. But in the PES/PCz case, the module gives a small amount of increase considered to PES. A similar effect has been reported in the literature, where the PANI-coated polyethylene ether phthalate (PET) textile module is reported to be better than uncoated PET [44] and is explained by filling the voids on the textile with PANI during the coating process. Since conductive polymers are harder materials than PES, this is an expected result. When Py and Cz copolymerized onto PES, the Young module reached the highest value. Considering that PPy has the lowest efficiency when comparing the polymer yields coated on PES, this may be the reason why the modulus is lowest in the PES/PPy case. The fact that the modulus of PES/PPy is lower than PES can be explained by the fact that PES interacts with the solvent during polymerization and becomes more flexible, as suggested in the literature [44]. Another reason might be the relatively brittle structure of PCz than PPy.

Structural, morphological, and mechanical analysis (IR, SEM, and mechanical test) suggested that PCz and PPy molecules have interactions to a certain extent with PES. Previous work [45] suggested that hydrogen-bonding interaction between the functional groups of template polymer or fiber and hydrogen atoms on the nitrogen of PPy chains plays a determining role in the formation of composites. In this study it is thought that similar to literature, hydrogen-bonding interactions between the oxygen atoms in carbonyl groups of PES and hydrogen atoms on nitrogen atoms of PPy or PCz are suggested to be the main interactions and simultaneously, the electrostatic attraction may play a secondary role on the formation of composites.

These interactions improved the adhesion of PCz and PPy on the PES surface and the coating stayed stable even after washing with ACN after polymerization and supported the existence of the coatings with characterizations. This shows that the polymer-PES interaction is sufficient enough to obtain the desired form.

## 4. Conclusion

By chemical polymerization of Cz with FeCl_3_, flexible, conductive PES/PCz were obtained on the PES surface. The effects of the wetting agent, oxidant type and concentration, solvent type, monomer concentration, and polymerization time were examined and the optimum condition was determined. Toluene, ACN, and water were used as solvents and the highest conductivity was obtained in the case of ACN. DBSA and PTSA were used as wetting agents, and DBSA was determined as the most suitable surfactant. Under these conditions, PES/P[Py-co-Cz] was obtained by the copolymerization of Cz with Py on the PES surface and was examined comparatively with PES/PCz and PES/PPy composites. The highest conductivity was obtained at 12.4 mS/cm for PES/PCz and 9.0 mS/cm for PES/P[Py-co-Cz]. FT-IR results proved that both Py and Cz were incorporated into the polymer structure. The stronger peaks in the FT-IR spectrum and the smoother surface formation in the SEM images support the same idea.

DMA measurements showed that Young’s modulus in the case of PES/PCz and PES/P[Py-co-Cz] were higher than that of PES. This can be explained by the interforce of polymer in the gaps of PES that contain many discontinuous fibers with interstices. This gap is filled with the PCz or P[Py-co-Cz] particles during the polymerization process; thus, the tensile properties are improved. as suggested in similar studies in the literature. On the other hand, the reason why it is higher than PES/PPy is the relatively brittle structure of PPy.

Considering that PPy has the lowest efficiency when comparing the polymer yields coated on PES, this may be the reason why the modulus is lowest in the PES/PPy case. The fact that the modulus of PES/PPy is lower than PES can be explained by the fact that PES interacts with the solvent during polymerization and becomes more flexible, as suggested in the literature. Although the conductivities of PES/PCz and PES/P[Py-co-Cz] obtained under the same conditions are close to each other, it has been observed that the mechanical property of PES/P[Py-co-Cz] has improved and this result makes the copolymer a suitable candidate in the field of smart textile technology such as gas and biomechanical sensors, antistatic applications and flexible supercapacitors.

## Figures and Tables

**Table 1:** Changes in conductivity with different waiting time (WT) and polymerization time, Cz = 0.01 M, FeCl_3_ = 0.6 M, ACN = 30 mL.

Procedure	WT in I min.	WT in II min.	WT in Cz, min.	WT in FeCl_3,_ min.	Repeating, times for III and IV	Total time, min.	Conductivity, S cm^−1^
			III	IV			
A	-	-	720	720	1	1440	no coating
B	30	30	60	10	8	270	12.42
C	30	30	120	10	8	540	0.015

**Table 2:** Content which provides optimal conditions for the solution used to wet the PES textile (I, II) and the copolymerization reaction takes place (III, IV).

Solution	I	II	III	IV
Content	0.02M DBSA 15 mL water	0.02M DBSA 15 mL ACN	0.005 M Cz 0.005 M Py 15 ml ACN	0.7 M FeCI_3_ 0.01 M DBSA 15 mL ACN

**Table 3:** Changes in conductivity of PES/PCz composites with solvents. Reaction time: 270 min (Cz = 0.01 M, FeCl_3_ = 0.6 M, solvent = 30 mL).

Solvent for Cz	Solvent for FeCl3	Conductivity, mS cm^−1^
Toluene	Toluene	0.64
Toluene	ACN	0.37
ACN+water	ACN	no coating
ACN	ACN	12.42

**Table 4:** The percentage yield (Y%) of the PES/PCz composites with changing the Cz and FeCl_3_ concentrations.

No	Cz, mol/L	FeCl_3_, mol/L	Y%
1	0.01	0.6	4.6
2	0.02	0.6	8.1
3	0.03	0.6	8.1
4	0.04	0.6	8.8
5	0.005	0.6	0.2
6	0.0025	0.6	0.4
7	0.009	0.6	2.2
8	0.008	0.6	1.8
9	0.007	0.6	1.4
10	0.05	0.6	9.5
11	0.06	0.6	7.2
12	0.01	0.7	2.5
13	0.01	0.5	1.4
14	0.01	0.4	2.1
15	0.01	0.3	1.5
16	0.01	0.2	2.0
17	0.01	0.1	1.0

**Table 5:** Young modules of PES, PES/PPy, PES/PCz, and PES/P[Py-co-Cz].

Polymer	Young Module, MPa
PES	431 [27]
PES/PPy	305
PES/PCz	467 [27]
PES/P(Py-co-Cz)	569
